# Development of a new Japanese version of the Clinical Impairment Assessment Questionnaire

**DOI:** 10.1186/s13030-020-00194-8

**Published:** 2020-08-31

**Authors:** Takeshi Horie, Maiko Hiraide, Shu Takakura, Tomokazu Hata, Nobuyuki Sudo, Kazuhiro Yoshiuchi

**Affiliations:** 1 Department of Stress Sciences and Psychosomatic Medicine, Graduate School of Medicine, The University of Tokyo, 7-3-1 Hongo, Bunkyo-ku, Tokyo, 113-8655 Japan; 2 Department of Psychosomatic Medicine, Kyushu University Hospital, 3-1-1, Maidashi, Higashi-ku, Fukuoka, 812-8582 Japan

**Keywords:** Eating disorders, Anorexia nervosa, Bulimia nervosa, Clinical impairment, Questionnaire

## Abstract

**Background:**

The Clinical Impairment Assessment questionnaire (CIA) is used to measure the severity of psychosocial impairment in patients with eating disorders. The purpose of the present study was to develop a new Japanese version of the CIA (CIA-J) and to evaluate its reliability and validity.

**Methods:**

We translated the sixteen items of the CIA into Japanese, back-translated them into English, and had them verified by a native English speaking professional editor. Participants were 152 Japanese-speaking patients (30.4 ± 10.6 years) under treatment for eating disorders and 173 healthy controls (29.5 ± 8.3 years). In addition to the CIA-J, the participants were asked to answer the Eating Attitudes Test (EAT26), The Positive and Negative Affect Schedule (PANAS), and the Hospital Anxiety and Depression Scale (HADS). We performed confirmatory factor analyses to evaluate the factor structure, calculated the Cronbach’s alphas of the CIA-J to assess the reliability, and calculated the correlation coefficients between the CIA-J score and those of EAT26, PANAS, and HADS to assess concurrent validity. We also used a Kruskal-Wallis test followed by Steel-Dwass test to compare the scores of the subtypes of eating disorders and the healthy control group.

**Results:**

A three-factor structure was obtained, similar to the original version. The Cronbach’s alphas of both the global and subscale scores of the CIA-J were high. The CIA-J had significant positive correlations with the EAT26, the negative affect subscale of the PANAS, and the HADS. The global and subscale scores for all subtypes of eating disorders were significantly higher than those of the healthy control group.

**Conclusions:**

The CIA-J was determined to be reliable and valid for assessing the severity of psychosocial impairment in patients with eating disorders.

## Background

Eating disorder patients have presumed “core psychopathology.” That is to say, they over-evaluate their shape, weight and control. Because these concerns about eating and its control prevent individuals from eating healthily, eating disorders have profound and specific effects on psychosocial functioning. The impairment of functioning secondary to eating disorder symptoms leads people to seek help and, consequently, improvement of functioning constitutes an important goal of treatment [1, 2]. Because clinically significant psychosocial impairment is a diagnostic requirement of eating disorders [3], we need to evaluate it correctly. The Clinical Impairment Assessment questionnaire (CIA) was developed in 2008 to measure such secondary impairment, and its reliability and validity have previously been confirmed [1]. This questionnaire measures three domains of impairment – personal (e.g. Made you feel critical of yourself), social (e.g. Stopped you going out with others), and cognitive (e.g. Made it difficult to concentrate). However, in Japan, there has not yet been any instrument whose reliability and validity has been confirmed for assessing clinically significant psychosocial impairment in eating disorder patients. The CIA has been the most widely used questionnaire to measure the severity of psychosocial impairment due to eating disorders [4]. Overseas it has been translated into various languages and, with the reliability and validity of these translated versions having already been confirmed, they are widely used for research and clinical use [5–11]. In order to investigate if the CIA can be used for patients with different cultural, socio-economic, and ethnic backgrounds, versions were developed for non-Western populations such as Fijian [6], Persian [9], and Singaporean (the majority of participants of Chinese ethnicity) [11]. However, there is yet to be a Japanese version of the CIA for which the reliability and validity have been confirmed. The aim of the present study was to develop a Japanese version of the CIA (CIA-J) and to evaluate its reliability and validity. Concerning validity, the following hypotheses are evaluated. CIA-J would show positive correlations with an eating-disorder related questionnaire and the scores would be higher in the patient group compared to the healthy group, as shown in previous studies on the development of a translated version of the CIA in other languages [10]. Additionally, CIA-J scores would show positive correlations with negative affect of PANAS and HADS because substantial impairments are related to negative affect, anxiety, and depressive symptoms.

## Methods

### Subjects

The patient group consisted of 152 female patients with eating disorders who fulfilled diagnostic criteria for anorexia nervosa or bulimia nervosa by the Diagnostic and Statistical Manual of Mental Disorders Fifth Edition (DSM-5) [3], who were 16 years or older, and who regularly received the treatment at The University of Tokyo Hospital or Kyushu University Hospital. The healthy group consisted of 173 healthy female participants 16 years or older who were recruited through a web survey by Macromill (http://monitor.macromill.com/), an internet research firm. The exclusion criteria for healthy participants were people with a body mass index (BMI) less than 17.5 kg/m^2^, an Eating Attitude Test (EAT-26) score greater than 20, or who self-reported receiving psychological or pharmaceutical treatment for any disease. All participants had adult level Japanese language competency. BMI is an index that represents a person’s degree of obesity and is defined as body weight (kg) divided by height (m) squared. The normal range of BMI is 18.5 to 25. EAT-26 is a questionnaire that measures the symptoms of anorexia nervosa. The group profiles are shown in Table 1. The study protocol was approved by the institutional review boards of The University of Tokyo and Kyushu University. The aims of this study were explained, and informed consent was obtained from all participants.

**Table 1:** Group profiles

	Patient group (*n* = 152) mean (SD)	ANR subtype (*n* = 54) mean (SD)	ANBP subtype (*n* = 58) mean (SD)	BN subtype (*n* = 40) mean (SD)	Healthy group (*n* = 173) mean (SD)
Age	30.4 (10.6)	30.7 (11.8)	31.3 (10.0)	29.0 (10.0)	29.5 (8.3)
BMI	17.6 (4.9)	15.0 (2.2)	16.0 (2.8)	23.5 (6.7)	20.9 (2.8)
EAT-26	29.3 (15.1)	24.4 (13.9)	32.5 (16.6)	31.3 (12.8)	4.4 (5.1)
PANAS (Positive affect)	26.9 (8.4)	28.7 (7.7)	25.5 (8.4)	26.7 (9.1)	28.2 (7.4)
PANAS (Negative affect)	36.0 (10.4)	33.4 (10.6)	36.4 (10.2)	38.8 (9.6)	26.0 (9.0)
HADS (Anxiety)	9.6 (4.7)	8.3 (4.8)	10.4 (4.9)	10.3 (4.0)	5.0 (3.2)
HADS (Depression)	8.4 (5.2)	6.5 (5.1)	9.5 (4.7)	9.4 (5.3)	4.9 (3.7)

*ANR* Anorexia nervosa restricting type, *ANBP* Anorexia nervosa binge-eating/purging type, BN = Bulimia nervosa, *EAT-26* 26-item version of the Eating Attitudes Test, *PANAS* Positive and Negative Affect Schedule, *HADS* Hospital Anxiety and Depression Scale

### Procedures

We first received permission to use CIA 3.0 from the publisher of the Japanese translation of a book by Kiriike [12] that included a Japanese translation of CIA 3.0 [13]. Then, we modified this version of the CIA to create a new Japanese version of CIA 3.0. All items of the new Japanese version were backtranslated into English and verified by a native English-speaking professional editor. Some items were modified according to the editor’s suggestions. We call this final Japanese version CIA-J. Participants were asked to complete the CIA-J, Eating Attitudes Test (EAT-26), The Positive and Negative Affect Schedule (PANAS), and Hospital Anxiety and Depression Scale (HADS).

### Measures

#### Cia-j

The CIA 3.0 is a 16-item questionnaire used to assess psychosocial impairment secondary to eating disorder features over the prior 28 days. Each item is rated on a 4-point Likert scale from 0 (“Not at all”) to 3 (“A lot”), with higher scores representing a higher severity of impairment. The original version of the CIA has three subscales (personal, social, and cognitive), and additionally produces a global score (0–48) that is designed to provide an overall index of the severity of current secondary psychosocial impairment. The clinical severity cut-off score of the original version is 16.

#### 26-item version of the eating attitudes test (EAT-26)

The EAT-26 was developed to measure the symptoms of anorexia nervosa. It includes three subscales: bulimia (six items), dieting (thirteen items), and oral control (seven items) [14]. The Japanese version of the EAT-26 has previously exhibited linguistic validity, acceptable internal consistency, and validity [15]. Although the EAT-26 is a questionnaire developed for the purpose of evaluating and screening AN, it consists of cognitions and behaviors regarding eating that are common to patients with eating disorders, which is why we used the questionnaire for analysis.

#### Positive and negative affect schedule (PANAS)

The PANAS was developed to measure positive and negative affect. It has twenty items: ten related to positive affect and ten related to negative affect [16]. The Japanese version of the PANAS was reported to have linguistic validity, acceptable internal consistency, and validity [17].

#### Hospital anxiety and depression scale (HADS)

The HADS was developed to detect states of anxiety and depression for patients with physical illness. It has fourteen items: seven related to anxiety and seven related to depression [18]. The Japanese version of the HADS was reported to have linguistic validity, acceptable internal consistency, and statistical validity [19].

### Statistical analyses

A global score of the original version of the CIA was calculated given that a minimum of 12 items had been rated. Any missing items were then prorated. For the healthy control group, computer-based data collection ensured that no items could be skipped. We performed confirmatory factor analysis (CFA) to evaluate the factor structure of the CIA-J. We examined two CFA models: the same three-factor model as the original version and a novel unidimensional model. To compare the fitness of the two models, we used the goodness of fit index (GFI), comparative fit index (CFI), root mean square error of approximation (RMSEA), and the Akaike information criterion (AIC). We calculated Cronbach’s alphas not only for the global score, but also for all three subscales of CIA-J to assess its internal consistency and reliability. Additionally, to assess concurrent validity, we calculated Spearman’s rank correlation coefficients between the scores on the CIA-J, which included the global score as well as the three subscale scores, and the EAT-26, HADS, and PANAS. The above-mentioned analyses were performed only in the patient group. In order to assess construct validity, we also performed a Kruskal-Wallis test followed by Steel-Dwass test to compare the scores for the subtypes of eating disorders with those of the healthy control group. Significance level was set to .05. In addition, a receiver operating characteristic (ROC) analysis was performed to determine the clinical significance cut-off point. All statistical analyses were conducted with JMP Pro 14.2.0 and Amos 22.0.

## Results

### CFA

**Fig. 1:**
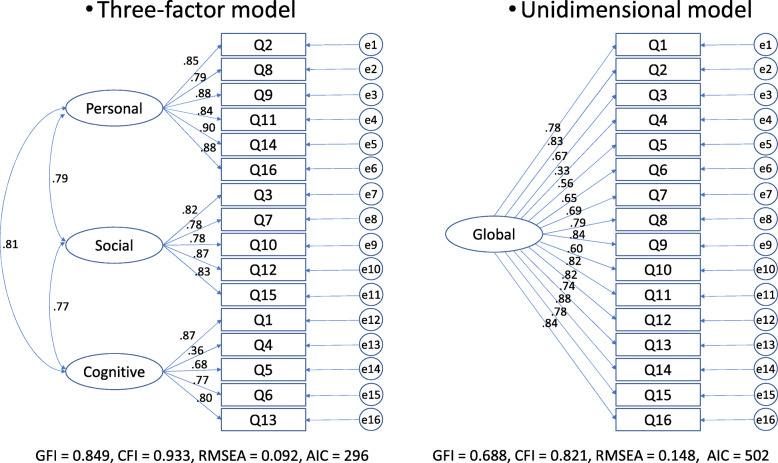
Confirmatory Factor Models. We drew path diagrams for the confirmatory factor analysis of the three-factor model and the unidimensional model with error terms e1-e16 and standardized parameter estimates. Below the path diagrams, we described the index of the fitness of each model

The results of the Confirmatory factor models are presented in Fig. 1. Similar to the original version, the three-factor structure exhibited a good fit (GFI = 0.85, CFI = 0.93, RMSEA = 0.092, and AIC = 296). Comparatively, the unidimensional model did not exhibit as strong of a fit (GFI = 0.69, CFI = 0.82, RMSEA = 0.15, and AIC = 502).

### Reliability

Cronbach’s alphas demonstrated good reliability for the three-factor model: 0.95 for the global CIA score, 0.94 for the personal subscale, 0.91 for the social subscale, and 0.83 for the cognitive subscale.

### Concurrent validity

The global and all subscale scores of the CIA-J had positive correlations with EAT26, negative affect of PANAS and the anxiety and depression subscales of the HADS, and global and personal subscale scores that had weak negative correlations with positive affect of PANAS (Table 2).

**Table 2:** Correlation coefficients with CIA-J for the patient group

	EAT-26 (Global score)	PANAS (Positive affect)	PANAS (Negative affect)	HADS (Anxiety)	HADS (Depression)
Global	0.60*	−0.23*	0.62*	0.64*	0.77*
Personal	0.59*	−0.25*	0.63*	0.65*	0.71*
Social	0.56*	−0.14	0.48*	0.50*	0.65*
Cognitive	0.44*	−0.21	0.54*	0.54*	0.69*

*p < .01

*EAT-26* 26-item version of the Eating Attitudes Test, *PANAS* Positive and Negative Affect Schedule, *HADS* Hospital Anxiety and Depression Scale

### Construct validity

The global and subscale scores of the CIA-J for all subtypes of eating disorders were significantly higher than those of the healthy control group. Additionally, the scores of the ANBP group and the BN group were higher than those of the ANR group, with the exception of the social subscale where there was no significant difference between the ANR group and BN group. In the comparison of the ANBP and BN groups, no significant differences were found between the global and subscale scores (Tables 3 and 4).

**Table 3:** Comparison of the three subtypes of eating disorders with the healthy controls (Multiple comparison)

	ANR (n = 54)	ANBP (n = 58)	BN (n = 40)	HC (n = 173)	Kruskal–Wallis Test		
	Median (Range)	Median (Range)	Median (Range)	Median (Range)	χ^2^ (df = 3)	ES (η^2^)	*p* value
Global	22 (0–45)	23.7 (0–44)	25.5 (0–45)	1 (0–35)	155.93	0.48	< 0.001
Personal	5.5 (0–18)	14 (0–18)	13 (0–18)	0 (0–15)	160.18	0.49	< 0.001
Social	2 (0–15)	7 (0–15)	5.5 (0–15)	0 (0–12)	144.84	0.44	< 0.001
Cognitive	1.5 (0–11)	4.5 (0–13)	4 (0–13)	0 (0–13)	92.34	0.28	< 0.001

*ANR* Anorexia nervosa restricting type, *ANBP* Anorexia nervosa binge-eating/purging type, *BN* Bulimia nervosa, *HC* Healthy controls, *ES* Effect size

**Table 4:** Comparison of the three subtypes of eating disorders with the healthy controls (Post hoc test)

	Post-hoc	BN vs. ANR		ANBP vs. ANR		BN vs. ANBP		ANR vs. HC		ANBP vs. HC		BN vs. HC	
	Steel-Dwass Test	p value	ES (r)	p value	ES (r)	p value	ES (r)	p value	ES (r)	p value	ES (r)	p value	ES (r)
Global	ANBP, BN > ANR > HC	0.008	0.33	0.001	0.35	n.s.	−0.01	< 0.001	0.48	< 0.001	0.65	< 0.001	0.58
Personal	ANBP, BN > ANR > HC	0.002	0.37	0.001	0.35	n.s.	0.00	< 0.001	0.49	< 0.001	0.65	< 0.001	0.71
Social	ANBP > ANR > HC, BN > HC	n.s.	0.22	0.005	0.32	n.s.	−0.10	< 0.001	0.47	< 0.001	0.70	< 0.001	0.60
Cognitive	ANBP, BN > ANR > HC	0.017	0.30	0.027	0.26	n.s.	0.05	< 0.001	0.28	< 0.001	0.53	< 0.001	0.50

*ANR* Anorexia nervosa restricting type, *ANBP* Anorexia nervosa binge-eating/purging type, *BN* Bulimia nervosa, *HC* Healthy controls, *ES* Effect size

### ROC analysis

**Fig. 2:**
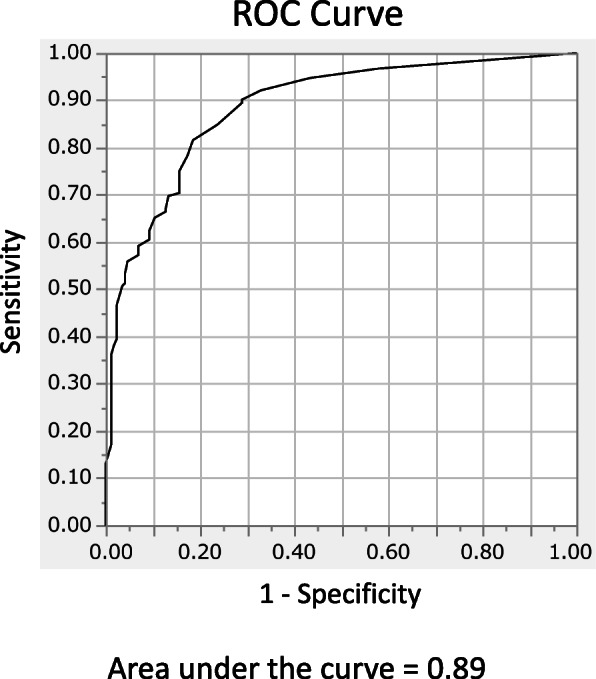
Receiver operator characteristic (ROC) curve analysis of the global CIA score for distinguishing positive from negative

The area under the curve of the ROC was 0.89, and the best cut-off point was a global CIA score of 6, which had a sensitivity of 81.6% and specificity of 81.5% (Fig. 2).

## Discussion

The purposes of this study were to produce a new Japanese version of the CIA (CIA-J) and to evaluate its reliability and validity using a sample from patients with eating disorders. From the results of CFA, similar to the original version [1], three subscales were confirmed: personal (6 items), social (5 items), and cognitive (5 items).

The Cronbach’s alphas of the global CIA-J and each of the three subscales were high, and these results are comparable with those in other studies with clinical samples [1, 10, 20]. This strongly suggests that the CIA-J expressed sufficient reliability.

The global and subscale scores of the CIA-J had significant positive correlations with the EAT26 scores, the negative affect subscale of the PANAS, and the HADS. In the original version of the CIA, the global score was significantly correlated with the global score of the EDE-Q and clinicians’ impairment ratings [1]. Although we used different questionnaires, we presented sufficient results to confirm concurrent validity. Regarding our test for construct validity, the CIA-J global score of the patient group was significantly higher than that of the healthy control group, which confirmed sufficient reliability and validity.

Although not reported in previous studies, we compared the scores by disease subtype. Among the patient group, the ANBP and BN groups had particularly high scores on CIA-J, which suggests that overeating and compensatory behaviors may be the major causes of secondary impairment. In a previous study, global CIA and subscale scores were significantly higher in patients who reported objective bulimic episodes, with the exception of the social subscale where there was no significant difference with or without objective bulimic episodes [10]. In this study as well, there was no significant difference between ANR and BN on the social subscale, which suggests that compensatory behaviors contributed more to social impairment than to overeating.

The clinical cut-off point for the CIA-J was 6, as compared to 16 in previous studies [1, 10]. If the cut-off point is set to 16, as in these previous studies, the sensitivity becomes 59.2% and false negatives increase. The reason for this finding may be that some patients had already improved their symptoms with treatment prior to study participation. In the study that reported the development of the original English CIA, both pre-treatment and post-treatment patients were included [1], and in another study, all groups of patients were pre-treatment [10]. In the future, we would like to verify the cut-off point by ROC analysis using a pre-treatment sample.

The limitations of this study include the following: First, we only performed a single administration of the CIA-J and have not yet assessed test-retest reliability. Second, we did not compare CIA-J scores from before and after treatment. Third, the original version of the CIA is designed to be completed after completion of the EDE-Q, allowing for comparison between the scores on the CIA, both original and translated versions, and the EDE-Q. Because the reliability and validity of the Japanese version of EDE-Q has not been proven, we could not use it for comparison.

## Conclusions

In the present study, we developed a novel Japanese version of the Clinical Impairment Assessment questionnaire (CIA-J). The CIA-J was found to be a reliable and valid instrument for use in assessing the severity of psychosocial impairment of female patients with eating disorders.

## Data Availability

Data cannot be shared publicly because datasets have ethical or legal restrictions for public deposition owing to inclusion of sensitive information from the human participants.

## Abbreviations

CIA: Clinical Impairment Assessment
BMI: Body Mass Index
EAT: Eating Attitudes Test
ANR: Anorexia nervosa restricting type
ANBP: Anorexia nervosa binge-eating/purging type
BN: Bulimia nervosa
CFA: confirmatory factor analysis
PANAS: Positive and Negative Affect Schedule
HADS: Hospital Anxiety and Depression Scale
GFI: Goodness of fit index
CFI: Comparative fit index
RMSEA: Root mean square error of approximation
AIC: Akaike’s information criterion
EDE-Q: Eating Disorder Examination Questionnaire
